# Elucidating Extracellular Vesicle Isolation Kinetics via an Integrated Off-Stoichiometry Thiol-Ene and Cyclic Olefin Copolymer Microfluidic Device

**DOI:** 10.3390/polym16243579

**Published:** 2024-12-21

**Authors:** Janis Cipa, Edgars Endzelins, Arturs Abols, Nadezda Romanchikova, Aija Line, Guido W. Jenster, Gatis Mozolevskis, Roberts Rimsa

**Affiliations:** 1 Institute of Solid State Physics, University of Latvia, 8 Kengaraga Str., LV-1063 Riga, Latvia; janis.cipa@cfi.lu.lv (J.C.); gatis.mozolevskis@cfi.lu.lv (G.M.); 2 Cellbox Labs LLC, 8 Kengaraga Str., LV-1063 Riga, Latvia; arturs.abols@biomed.lu.lv; 3 Latvian Biomedical Research and Study Centre, Ratsupites Str. 1, LV-1067 Riga, Latvia; edgars.endzelins@biomed.lu.lv (E.E.); nadezda.romanchikova@biomed.lu.lv (N.R.); aija@biomed.lu.lv (A.L.); 4 Department of Urology, University Medical Center Rotterdam, Dr. Molewaterplein 40, 3015 GD Rotterdam, The Netherlands; g.jenster@erasmusmc.nl

**Keywords:** PDMS-free, microfluidics, extracellular vesicles, binding kinetics, immunomagnetic separation

## Abstract

Extracellular vesicles (EVs) are promising biomarkers for diagnosing complex diseases such as cancer and neurodegenerative disorders. Yet, their clinical application is hindered by challenges in isolating cancer-derived EVs efficiently due to their broad size distribution in biological samples. This study introduces a microfluidic device fabricated using off-stoichiometry thiol-ene and cyclic olefin copolymer, addressing the absorption limitations of polydimethylsiloxane (PDMS). The device streamlines a standard laboratory assay into a semi-automated microfluidic chip, integrating sample mixing and magnetic particle separation. Using the microfluidic device, the binding kinetics between EVs and anti-CD9 nanobodies were measured for the first time. Based on the binding kinetics, already after 10 min the EV capture was saturated and comparable to standard laboratory assays, offering a faster alternative to antibody-based immunomagnetic protocols. Furthermore, this study reveals the binding kinetics of EVs to anti-CD9 nanobodies for the first time. Our findings demonstrate the potential of the microfluidic device to enhance clinical diagnostics by offering speed and reducing manual labor without compromising accuracy.

## 1. Introduction

Over the past decade, extracellular vesicles (EVs) have been of key interest as a biomarker for the detection of complex diseases such as cancer and neurodegenerative and cardiovascular diseases [1]. EVs are heterogeneous, plasma membrane-bound vesicles secreted by virtually all cell types that contain various cargo messenger molecules and can trigger a variety of cellular responses. Their diagnostic potential, particularly in liquid biopsy applications, has garnered increasing attention for clinical application [2,3]. EV-based analysis offers the potential for rapid disease detection, the monitoring of disease progression, and assessing drug effectiveness, ultimately improving patient care [4,5,6]. However, the implementation of EV-based diagnostics in clinical settings has been considerably impeded, primarily attributed to the lack of robust, efficient, and reproducible methodologies for the isolation of specific EV subpopulations. This issue is further amplified by their broad size distribution, spanning from 30 nm to 2000 nm, which poses substantial challenges for their analysis using standard laboratory assays [1].

Various methods exist for separating and analyzing EVs, such as ultracentrifugation [7], size exclusion chromatography [8], precipitation [9], and others [10]. However, the ongoing challenge lies in obtaining specific EV subpopulations that are separated from cell debris and nonspecific EVs, and are enriched to perform quantitative downstream analysis. As such, immunomagnetic isolation represents the gold standard method due to efficient, fast, scalable, and specific extractions of EVs [11,12,13,14,15,16,17]. Furthermore, immunomagnetic isolation has been used for different purposes such as bacteria capture and DNA extraction [18], metabolite capture [19], EV separation [7], and others [20].

Building on existing methods, microfluidic devices represent a notable advancement in EV separation as they have shown promise with better enrichment compared to traditional methods like ultracentrifugation [7], along with higher throughput [21], reduced sample volume [22], and reduced labor intensity. Notably, Wang et al. demonstrated a microfluidic device’s efficiency in single-chip analysis using magnetic particles and Raman spectroscopy, distinguishing between cancer patients and healthy individuals within an hour, where the EV capture efficiency was 72.5% [23]. A potential limitation of the current presented EV separation devices is the slow flow rate of 0.3 µL/min, which for a typical clinical sample size of at least 100 µL and a gold standard limit of detection of 12–15 IU/mL would take 5.5 h [24].

Given the prevalent surface markers on the surface of EVs, antibody capture mechanisms have been heavily utilized in the presented microfluidic and macroscale systems [25]. Recently, however, single-domain antibodies, typically derived from animals, have surged as an alternative to existing antibody-based capture [26]. Ref. [27] Nanobodies offer increased stability and better permeability due to smaller sizes retaining the same capture mechanisms as the antibodies; thus, nanobodies have emerged as a potential alternative in the diagnostics sector [27].

Despite several promising advantages of microfluidic devices, the applicability of microfluidics in EV diagnostics has been severely hindered due to materials typically used in device fabrication. Microfluidic devices typically use polydimethylsiloxane (PDMS) as their primary material; while PDMS is excellent for prototyping, it presents limitations for widespread adoption in EV analysis due to challenges in scaling for mass production and its tendency to absorb lipophilic molecules [28,29]. Moreover, in applications involving biological samples, the imperative for sterility necessitates the use of single-use devices, rendering PDMS economically impractical due to high costs.

In response to these constraints, alternative materials are being actively explored, with a focus on thermoplastics and other moldable substrates. Among the promising candidates are cyclic olefin copolymer (COC) and off-stoichiometry thiol-ene polymer (OSTE), where both materials show promise in reducing hydrophobic molecule absorption and offering structural versatility [29,30,31,32]. COC has been widely used as a biocompatible and transparent thermoplastic in different microfluidics applications [33], whereas OSTE materials have been incorporated or used separately for applications such as synthetic paper for enrofloxacin detection [34], spider silk fabrication [35], and as a channel for photonic biosensors [36], among others. Yet, to the best of our knowledge, OSTE-COC devices have only been fabricated for organs on chips [37] and EV separation based on flow field–flow fractionation [38] rather than a microfluidic EV sample preparation tool.

To advance EV extraction techniques and further integrate microfluidic biosensing applications, this study encompasses a comprehensive approach, covering the entire workflow from device fabrication to biological application. By introducing a proof-of-principle EV extraction device that integrates mixing and magnetic particle separation functions on a PDMS-free chip, we demonstrate advancements in assay turnaround time and process readiness for the fabrication of derivative designs. This novel approach not only improves the scalability of EV extraction but also offers a practical and versatile platform for testing with liquid biopsy samples containing EVs, paving the way for potential clinical applications.

## 2. Materials and Methods

### 2.1. Device Fabrication

OSTE-COC microfluidic device computer-aided design models were created for the photomask and 3D-printed mold in SolidWorks. The photomask was fabricated using standard lithography with positive AZ1518 resist (MicroChemicals, Ulm, Germany), and a direct laser writer (375 nm, Heidelberg µPG, Mittelgewannweg, Germany) was used for a 25 × 76 × 1 mm^3^ patterned glass slide. After development, a 50 nm Al_2_O_3_ layer was deposited via atomic layer deposition (ALD Savannah) to protect the mask and ensure the repeatability of the mask.

A double-negative mold was 3D-printed (Zortrax, Olsztyn, Poland, basic white/ivory) using a resin 3D printer (Zortrax inkspire), where the layer thickness was 50 µm and pixel size was 50 × 50 µm^2^. A post-printing mold was exposed with UV light for 810 s (12 J/cm^2^) and cured for at least 48 h at 60 °C. A post-curing negative mold was made from soft, 2-component silicone, Qsill 216 (CHT smart chemistry, Tübingen, Germany).

The device substrate (140 µm, TOPAS, microfluidic chip shop) was O_2_ plasma-treated (O_2_ 700 sccm, 700 W, 5 min, 2.45 GHz, PVA TePla gigabatch 360M, Wettenberg, Germany ) with a COC sheet used to cap the Qsill mold. Microfluidic devices were fabricated using reaction injection molding of OSTE 220 (Mercene Labs, Ostemer, Stockholm, Sweden) using Al jig. Post-compression OSTE 220 was injected into the Qsill mold cavity at 800 mBar (Elveflow OB1 flow controller, Paris, France). After OSTE 200 injection, the device was exposed using ND33 and I-line filter (Mask aligner Suss MA6, Garching, Germany) to decrease exposure intensity to 6 mJ/(cm^2^s), with a final dose of 108 mJ/cm^2^.

After exposure, the device was developed in acetone in an ultrasonic bath (80 kHz, 50%, Elmasonic P, Singen, Germany) for 75 s, where the reaction was subsequently stopped by isopropanol and N_2_ blow-dried. A post-development bake was performed at 60 °C for 3 min to reduce mechanical stress and allow for better layer bonding. Before bonding the top part of the device, the COC slide was treated with O_2_ plasma (700 sccm O_2_, 700 W, 3 min).

Finally, both layers were aligned, pressed together, and exposed to a 4000 mJ/cm^2^ dose. Subsequently, the device was cooled down for a few minutes, resulting in a hermetically sealed device, with a burst point exceeding 400 µL/min.

### 2.2. Flow Controls

Microfluidic flow was controlled using 5 mL syringes and syringe pump (ISPLab02 DKinfusetek, Hvidovre, Denmark). The connection between different device modules was ensured using 800 μm diameter polytetrafluoroethylene (PTFE) tubing, 800 µm diameter Masterflex C-Flex tubing (Darwin microfluidics, Paris, France), and polypropylene Luer ports (ChipShop, Jena, Germany).

### 2.3. Hollow-Fiber Bioreactor Culture of LNCaP Cells

A C2011 high-flux PS hollow-fiber cartridge (Fibercell, New Market, MD, USA) was pre-coated with 0.5 mg human fibronectin and inoculated with 2.76 × 10^8^ LNCaP cells. During the colonization phase, 125 mL LNCaP flask culture medium (RPMI + 2 mM sodium pyruvate + 2 mM L-glutamine + 10% FBS), supplemented with additional glucose up to 4 g/L, was recirculated through the cartridge. Circulating media was exchanged based on daily glucose consumption readings, the volume was gradually increased, and the composition gradually switched to DMEM/F12 with the same supplements. Once reaching 1000 mg daily glucose consumption, FBS was substituted by 10% CDM-HD serum replacement (Fibercell) and daily conditioned media harvesting from the extracapillary space (ECS) was commenced. The conditioned media was pre-processed by sequential centrifugation (5 min at 300× *g*, 30 min at 3000× *g*) and stored at +4 °C until isolation of EVs.

### 2.4. Isolation of EVs

EVs were isolated from the conditioned cell culture media by size exclusion chromatography (SEC) using qEV10 35 nm SEC columns (Izon, Bellarie, TX, USA) according to the manufacturer’s guidelines. Prior to loading onto columns, the pre-processed media samples from up to 4 sequential day harvests were pooled together, filtered through 0.2 μm syringe filters, and concentrated down to 10 mL using Amicon-15 100 kDa ultrafiltration tubes (Merck KGaA, Darmstadt, Germany). The particles in each 5 mL SEC fraction were analyzed with Zetasizer Nano (Malvern), and the EV-containing ones (usually 4 fractions) were pooled together and concentrated using Amicon 3 kDa ultrafiltration tubes (Merck KGaA, Darmstadt, Germany). EV preparations were aliquoted and stored at –80 °C.

### 2.5. EV Concentration Measurements

The size distribution and concentration of EVs were assessed using nanoparticle tracking analysis (NTA) with the NanoSight NS300 instrument (Malvern, Malvern, UK). Briefly, EV samples were diluted 500–2000-fold in PBS to achieve a concentration range of 1 × 10^8^ to 1 × 10^9^ particles/mL and introduced into the flow cell using a 1 mL syringe. Five 60 s videos were recorded at camera level 14, with the sample manually advanced between recordings. Particle tracking was conducted at a detection threshold of 4–5. Protein concentration was determined by Pierce BCA protein assay (Thermo Fisher, Waltham, MA, USA).

### 2.6. Transmission Electron Microscopy

First, 10 µL of EV suspension was applied onto a 300-mesh carbon-coated copper EM grid and incubated for 5 min. The grid was negatively stained with 1% uranyl formate solution for 60 s. After air-drying, examination was carried out using a JEM-1230 microscope (JEOL, Peabody, MA, USA).

### 2.7. Anti-CD9 Nanobody Production

DNA sequence encoding nanobody recognizing CD9 was generously provided by Prof. Guido Jenster. The sequence was further engineered to contain C-terminal His_6_ and HA affinity tags, and adapted for cloning into pET-26b(+) periplasmic expression plasmid. The gene synthesis and cloning into the plasmid were outsourced to BioCat GmbH, Heidelberg, Germany.

BL21 (DE3) *E. coli* cells were subjected to transformation with the Nb-encoding plasmid. After confirming the periplasmic presence in WB with an antibody against His_6_ tag (MA1-21315, Thermo Fisher; in 1:3000 dilution), anti-CD9 Nb was purified from 540 mL of transformed culture. In brief, induced cells were pelleted at 6500× *g* for 15 min, +4 °C. The pellet was resuspended into 240 mL 30 mM Tris-HCl pH 8.0 + 20% sucrose and brought to 1 mM EDTA, then slowly stirred for 10 min at RT. The cells were pelleted again by centrifugation at 6500× *g*, 15 min, +4 °C, resuspended into 240 mL ice-cold 5 mM MgSO_4_, and slowly stirred for 10 min while maintaining +4 °C. Next, the shocked cells were pelleted at 6500× *g*, 15 min, +4 °C, and the supernatant was collected. The supernatant (periplasmic lysate) was brought to 150 mM NaCl, 2 mM MgCl_2_, 20 mM imidazole, pH 7.5 and filtered through 0.2 μm before loading onto 5 mL HisTrap™ Fast Flow columns (GE17-5255-01, GE Healthcare, Chicago, IL, USA) for purification using ÄKTAprime plus (Cytiva) chromatography system. In short, each column was equilibrated with 5–10 volumes of 1× Binding Buffer (150 mM NaCl, 2 mM MgCl_2_, 20 mM imidazole, pH 7.5), followed by application of the sample. The column was washed first with 1× high-salt buffer (20 mM HEPES, pH 7.5; 500 mM NaCl; 20 mM imidazole), and then with 1× NH7.5 buffer (2 mM HEPES, pH 7.5; 100 mM NaCl) + 20 mM imidazole until the absorbance reached the baseline. The purified Nb was eluted in 1× NH7.5 buffer (2 mM HEPES, pH 7.5; 100 mM NaCl) + 400 mM imidazole. Approximately 7 mL of the elute was collected and subjected to buffer replacement and concentration on Amicon-15 3 kDa spin columns (5000× *g*, +4 °C). Nanobody samples were aliquoted for storage at −20 °C into protein low-bind tubes (Eppendorf). Nb concentration was determined by Pierce BCA protein assay (Thermo Fisher).

### 2.8. EV Capture ELISA

Anti-CD9 nanobody affinity towards native CD9 on EV samples from multiple cell lines was assessed in exosome ELISA using PS Capture™ Exosome ELISA Kit (297-79201, FUJIFILM Wako, Tokyo, Japan) according to the manufacturer’s protocol. All EVs were used in amounts of 2 × 10^8^ pts per ELISA well. The nanobody was tested in increasing concentrations from 2 to 20 μg/mL and secondary anti-His6-HRP (MA1-21315-HRP, ThermoFisher) was used at a 2 μg/mL concentration.

### 2.9. Anti-CD9 Magnetic Particles

Anti-CD9 magnetic particles (MPs) were prepared by loading each 1 mg of Pierce anti-HA magnetic beads (Thermo Fisher) with 20 μg anti-CD9 nanobody featuring a C-terminal HA affinity tag. The loading was performed for 30 min at room temperature on a tube rotary mixer (15 RPM), and afterwards any excess nanobody was removed from the reaction by washing 4× with PBS + 0.1% BSA + 0.05% Tween-20.

### 2.10. EV Capture to Anti-CD9 Magnetic Particles

Anti-CD9 bead performance was initially characterized by setting up test tube EV capture reactions between 0.6 mg magnetic particles and 6 × 10^9^ LNCaP EV pts in 0.6 mL PBS + 0.1% BSA, which were rotated (10 RPM) either for 1 h at room temperature or for 3 h at +4 °C. Afterwards, any unbound EVs were removed by washing the MPs 3× with 1 mL PBS + 0.1% BSA + 0.05% Tween-20 and once with 1 mL PBS. Afterwards, all the supernatant was removed and the MPs were heated for 5 min at 95 °C in 20 μL of non-reducing Laemmli buffer. The supernatant containing lysated EVs was then collected and directly analyzed by Western blot (10 μL per each lane) with HRP-conjugated antibody against CD63 (Novus Biologicals, Centennial, CO, USA, NBP2-34779H, 1:2000 dilution) and the novel nanobody against CD9 in conjunction with secondary anti-His_6_—HRP (Thermo Fisher, MA1-21315-HRP, 1:1000 dilution).

### 2.11. Sample Flow Control and Washing

EV sample and surface-modified magnetic particles were loaded in separate syringes or tubing, where a 25 µL air gap was used to separate samples from the wash buffer, which was sequentially used after sample inflow. ON-Chip washing was conducted by flushing the device with 3 mL of PBS + 0.1% BSA + 0.05% Tween-20 solution. In case cycling was used, the withdrawal flow rate was reduced to 100 µL/min to prevent air creep in the system. To extract the sample, the magnet array was removed and the MPs containing bound EVs were eluted. For comparison, a standard benchtop EV capture protocol was performed by reacting the same samples in a 2 mL test tube on a rotary mixer (15 RPM) for 50 min, and washing 4× with 1 mL of PBS + 0.1% BSA + 0.05% Tween-20.

### 2.12. Western Blot

For Western blot (WB) analysis, 10 μL of lysate in either reducing or non-reducing (for anti-tetraspanin antibodies) Laemmli was loaded per each lane of a 10% SDS-PAGE gel. Following the separation at 150 V, the proteins were transferred onto 0.45 µm nitrocellulose membranes, which were subsequently blocked using 10% (w/v) fat-free milk for 1 h at room temperature. The membranes were then incubated with primary antibody (Table S1) overnight at +4 °C and 60 RPM, and washed 4 times with TBS + Tween-20 (0.05%, 0.1%, 0.1%, 0.05%). The incubation with secondary antibody (Table S1) was conducted for 1 h at room temperature 60 RPM, followed by washing as previously described. Detection of immunoreactive bands was carried out using the Amersham™ ECL Select™ Western Blotting Detection Reagent kit (GE HealthCare) and pictures were taken using a Nikon d610 dSLR body (Nikon, Tokyo, Japan) with Sigma 35 mm f/1.4 DG HSM Art lens (Sigma, Kawasaki, Japan). To acquire numerical band integrated density values, ImageJ software (version 1.54g) was used. To account for differences in compared WB membranes, total EV quantity was used to normalize the signal intensity and is indicated on the graphs as total EV quantity or ctrl. +. When necessary, the membrane was stripped in a buffer containing 1.5% glycine, 0.1% SDS, 1% Tween-20, pH 2.2, blocked again, and re-probed with a different set of antibodies as previously described.

## 3. Results and Discussion

### 3.1. Microfluidic Device

**Figure 1:**
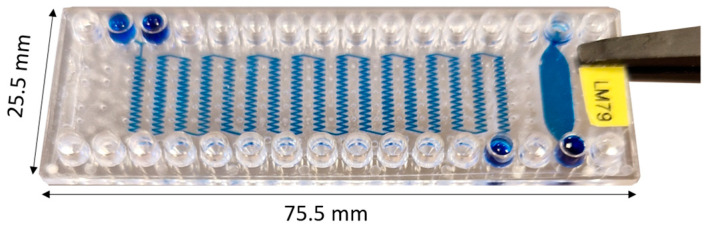
Fabricated OSTE-COC device, macro-image.

To facilitate the mass production of a sterile device, a passive zigzag mixing design was chosen [39]. Its modularity allows for adjustable length based on mixing needs, while its 2D structure supports efficient manufacturing via injection molding further in the project, but also CNC milling, and laser ablation that allows for rapid design prototyping. Additionally, the lack of active components simplifies assembly, thus enhancing scalability and cost-effectiveness. Device geometry (Figure S1) was optimized based on our results of mixing index testing, following methodology described by Hashmi [40] (Table S2), where flow rate was tested from 50 µL to 200 µL and points to a strong correlation between increasing flow rate and increases in mixing (*p* = 2.57 × 10^8^). Secondly, decreasing channel size from 300 µm to 200 µm showed a statistically significant increase in relative mixing index (*p* = 9 × 10^−5^). Thirdly, no significant effect of varied bend angle in the range of 75 to 81 degrees (*p* = 0.278) can be seen, with analysis performed at the 95% confidence interval, where similar results were reported by Cosentino et al. for multiple bends in a passive micromixer [41]. As such, a device with 200 × 200 µm channels and 764 mm length was fabricated using OSTE 220 material (Figure 1). The resolution of the smallest corner radius was 20 µm, and a burst point exceeded 400 µL/min as tested by operating devices using DI water. Furthermore, the finished device consisted of an OSTE layer compressed between two COC layers; thus, the device had excellent optical properties due to COC visible light transparency [42]. A similar device fabrication process has been previously reported by Sandström N. [43], but in our case, a COC sheet and COC slide with luer connections were used, which allows for simple connections and the potential for design scalability. Furthermore, the fabrication protocol builds on our previous work [31] by decreasing the feature size down to 20 µm and improving the fabrication speed, as this fabrication method allows us to remove thermal treatment; thus, the ready device can be made in under 1 h.

**Figure 2:**
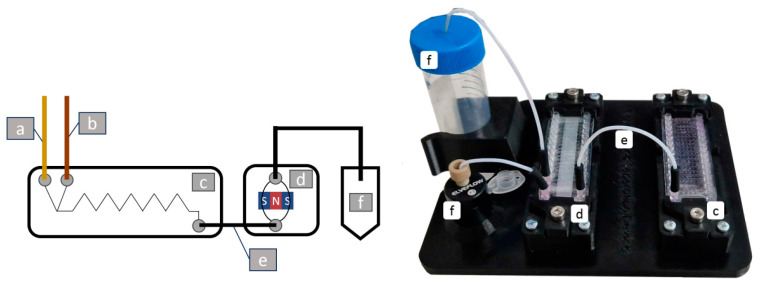
Schematic of the microfluidic device incorporating MP injection tubing (a), sample injection tubing (b), mixing device (c), MP separation module (d), tubing for cycling or shorter tubing for connection to a sample collection container (e), sample collection container (f) on the left and macro-image on the right.

To test magnetic particle and EV interactions in fabricated microfluidic devices, an experimental setup was created using a fused deposition modeling (FDM) 3D printer, as seen in Figure 2. This setup includes two identical OSTE-COC microfluidic devices with a volume of 27 µL and 510 bends (see Figure 2c for mixing and magnetic separation chamber), with surface area of 55 mm^2^, as shown in Figure 2d. MP separation was conducted based on our previous publication using a 4 mm wide magnetic separation chamber (Figure S2) [44]. Four magnetic configurations were tested: a single N52 magnet under the channel Figure S3a; two N52 magnets, both under and above the channel Figure S3b; 18 (8 × 5 × 5 mm^3^) N45 magnets stacked in alternating polarity (Figure S3c and in Halbach array Figure S3d). The optimal configuration was determined to be alternating polarity, as it was a stable configuration with a retention of over 95% of the magnetic particles at a 200 µL/min flow rate [44]. The magnet array was placed in a 3D-printed jig to fix the device and decrease the air gap between the device and magnet array (Figure 2d).

### 3.2. Hollow-Fiber Bioreactor Production of Extracellular Vesicle Standards

**Figure 3:**
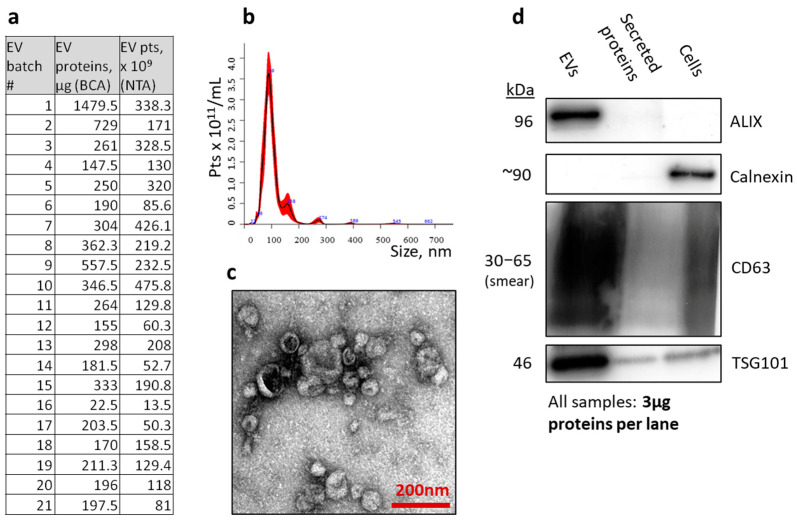
Extracellular vesicle (EV) production from LNCaP hollow-fiber bioreactor culture. EV protein amounts and particle counts in all batches purified during 71-day timeframe (**a**). Representative EV particle size distribution by nanoparticle tracking analysis (**b**). Representative transmission electron microscopy image (**c**). Characterization of EV markers by Western blot (**d**).

For testing EV capture in the microfluidic device, a sufficient amount of standardized EV preparation was necessary. For this purpose, a C2011 high-flux PS hollow-fiber cartridge (Fibercell) with 2.76 × 10^8^ LNCaP cells was inoculated and harvested daily from the extracapillary space (ECS) as soon as optimal colonization was achieved. EVs from combined 3- or 4-day harvests were purified by size-exclusion chromatography (SEC) and measured by nanoparticle tracking analysis (NTA) (Figure 3b) and the BCA protein assay. In summary, this bioreactor culture yielded 3.92 × 10^12^ EV particles (6.9 mg EV proteins) during a 71 day timeframe (Figure 3a). Select EV batches were also characterized by transmission electron microscopy (TEM), which indicated a polydisperse mixture of predominantly sub-100 nm particles (Figure 3c). Western blot (WB) analysis on select EV batches, compared to cell lysates obtained from the same bioreactor culture, and ECS-secreted proteins, were consistent with highly pure EV preparations, revealing enrichment in EV-characteristic markers (ALIX, CD63, and TSG101), and no presence of endoplasmic reticulum marker Calnexin, which was used as a negative control (Figure 3d).

### 3.3. Magnetic EV Capture Particles Featuring Novel Anti-CD9 Nanobody

**Figure 4:**
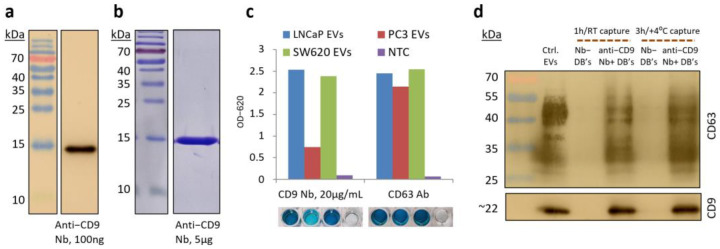
Anti-CD9 nanobody (Nb) quality control and performance assessment. Western blot detection of His_6_-tagged anti-CD9 Nb in purified Nb preparation (**a**). Coomassie staining of total proteins in purified Nb preparation (**b**). PS Capture™ Exosome ELISA analysis indicating an exceptional extracellular vesicle (EV) capture by anti-CD9 Nb at 20 μg/mL as compared to the anti-CD63 control (**c**). WB demonstrating LNCaP EV capture performance of anti-CD9 nanobody-coated Dynabeads^TM^ under various capture conditions (**d**).

The DNA sequence for the nanobody (Nb) recognizing CD9, and abundant EV surface markers, were kindly provided by Prof. Guido Jenster. Here, we further modified the sequence to encode C-terminal His_6_ and HA affinity tags to facilitate downstream purification, detection, and surface immobilization workflows, expressed it in BL21 (DE3) *E. coli* strain, and purified it from its periplasmic lysate by IMAC chromatography. Ultimately, 540 mL of induced BL21 (DE3) culture (OD_540_ = 6.8) yielded 18.1 mg of purified product (BCA protein assay). The product was then confirmed in WB with anti-His_6_ antibody (Ab) to be consistent with the expected 16 kDa molecular weight of the anti-CD9 Nb (Figure 4a) and further assayed for purity by Coomassie staining, which indicated no other molecular weights present (Figure 4b). PS Capture™ Exosome ELISA indicated a strong affinity towards EVs obtained from multiple cell lines (LNCaP, PC3, and SW620) starting from 2 μg/mL anti-CD9 Nb concentration. The signal intensity was comparable to the control antibody (against CD63) of the ELISA kit at the Nb concentration of 20 μg/mL when tested against EVs from CD9-positive lines (LNCaP and SW620) and, as expected, there was a decreased reactivity against EVs obtained from the CD9-low PC3 line (Figure 4c). Finally, we loaded Pierce anti-HA magnetic particles (Dynabeads™) with 20 μg of anti-CD9 Nb per each 1 mg of MPs and tested their ability to capture LNCaP EVs at 1 mg/mL bead concentration under different reaction conditions. WB analysis against CD63 and CD9 EV markers in the lysates from the capture MPs after the reaction, both when reacted for 1 h at room temperature and for 3 h at +4 °C indicated nearly complete EV capture compared to the same amount of EVs in the positive control (Figure 4d). Additionally, no captured EVs were detected if the capture MPs were prepared without Nb, indicating high capture specificity (Figure 4d).

### 3.4. Flow Rate Sweep for Anti-CD9 Magnetic Particles

**Figure 5:**
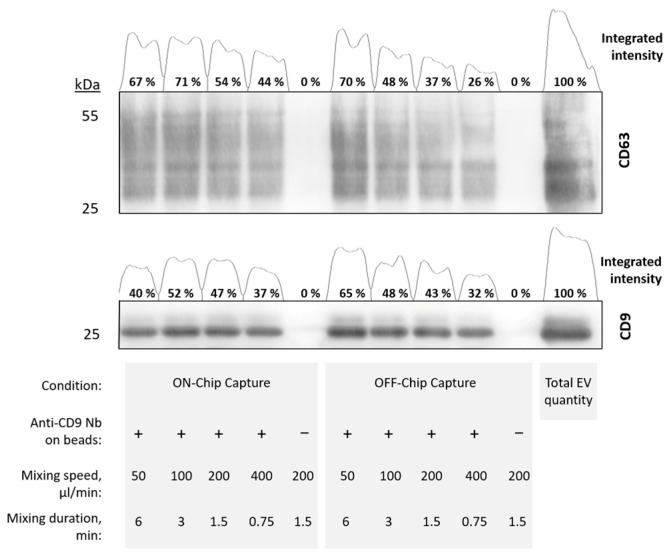
EV and anti-CD9 nanobody-conjugated MP mixing in OSTE-COC device. MP retention was conducted either ON-Chip or OFF-Chip at different flow rates. Numerical values are normalized relative to the total initial quantity of extracellular vesicles used in each capture reaction (as indicated in the last Western blot column). Anti-CD9 specificity of MPs was tested by including MPs not containing nanobody (denoted by −) in reaction with EVs.

To evaluate the efficiency of the MP separation in the microfluidic device compared to traditional lab assays, a flow rate sweep was conducted under conditions relevant to sample viscosity. LNCaP EVs were utilized in the microfluidic device, employing 1 µm diameter magnetite core MPs conjugated with anti-CD9 nanobody for EV capture. Following EV capture by MPs, EV-containing MPs were separated from the sample, either ON-Chip or in a tube, with subsequent washing performed either ON-Chip or OFF-Chip (in tube). For ON-Chip separation, MPs were washed with PBS buffer solution (PBS + 0.1% BSA + 0.05% Tween-20) at 200 µL/min and then collected in a new container. OFF-Chip separation followed a standard procedure where EV-containing MPs were retained using a benchtop magnet, followed by triple washing on the magnet. EV-containing MPs were heated in 1× Laemmli sample buffer for 5 min at 95 °C, followed by MP removal with a benchtop magnet, and loading the supernatant on SDS-PAGE gel for Western blot analysis. Figure 5 illustrates the relative intensity of the CD63 bands, reflecting the EV capture efficiency across both ON-Chip and OFF-Chip workflows.

The Western blot results from Figure 5 reveal that at 50 µL/min using OFF-Chip MP retention, the intensity accounts for 70% and 65% of the total EV count, which surpasses that of the ON-Chip MP retention at 67% and 40% for CD63 and CD9, respectively. This discrepancy could be attributed to differences in setup configurations. In the ON-Chip setup, MP retention occurs on the chip, ensuring that all non-captured EVs pass through magnetically bound MPs and are subsequently discarded. In contrast, OFF-Chip retention allows for post-mixing interactions within the container, potentially extending incubation time and subsequently enhancing signal intensity. This implies that although the ON-Chip setup provides a more accurate representation of the actual interaction time, it yields a diminished signal due to effectively reduced incubation time.

Moreover, linear regression analysis indicates that the decrease in CD63 intensity is marginally significant for both systems (*p* = 0.058 for ON-Chip and *p* = 0.10 for OFF-Chip). For ON-Chip CD9 retention, a flow-independent result is observed (*p* = 0.49), while for OFF-Chip, a marginally significant intensity decrease is noted (*p* = 0.091). This suggests that ON-Chip capture produces more consistent results than OFF-Chip capture (Figure S4). To enhance binding efficiency and elucidate the binding process, multiple cycles through the mixer were introduced, thereby increasing interaction within the mixing module.

### 3.5. Proof-of-Principle EV Sample Preparation Unit

To assess binding kinetics and demonstrate the performance of a proof-of-principle sample preparation unit, a complete system comprising an integrated mixing module and magnetic separation module was tested and compared to a standard lab assay in a semi-automatic operation setting.

**Figure 6:**
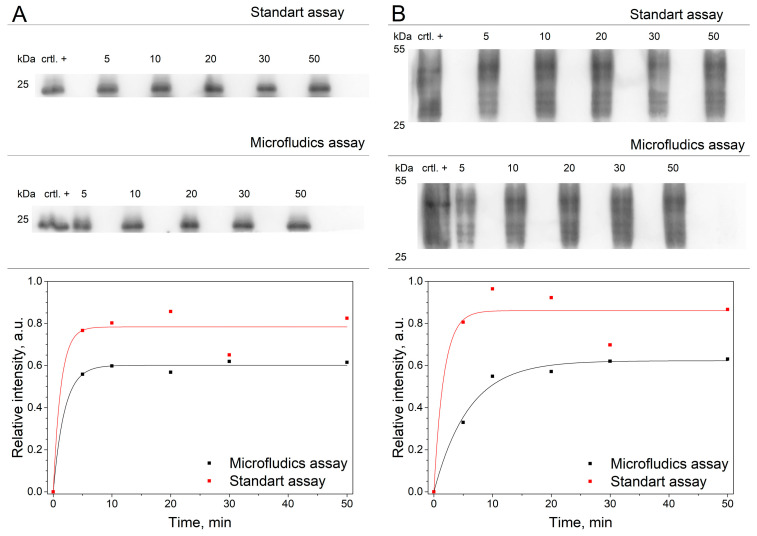
Western blot analysis showing the efficiency of EV capture. Anti-CD9 EV capture and CD9 detection (**A**) and WB results for anti-CD9 EV capture and CD63 detection (**B**) where the top figure is WB image, and each data point is an integrated value relative to EV marker signal intensity.

Analysis of EV binding kinetics was conducted using LNCaP EVs mixed with 1 μm MPs conjugated with anti-CD9 nanobodies, with a total sample volume of 300 μL and a flow rate of 150 μL/min. The EV sample and MPs were cycled 1 to 10 times to enhance EV binding. Moreover, the process was semi-automated by preloading the sample and buffer solution for post MP retention washing in the syringe pumps. Following the mixing of LNCaP EVs with anti-CD9 MPs in a microfluidic device, magnetic separation was used to separate post-EV capture MPs from reaction mix. ON-Chip washing was performed subsequently using a flow rate of 150 µL/min. Post-washing, the MP captured EVs were subjected to heat treatment in 1× Laemmli sample buffer for EV lysis and the supernatant was loaded directly onto SDS-PAGE gel for subsequent Western blot analysis. Simultaneously, a standard benchtop EV capture reaction with the same MPs were performed for comparative purposes (Figure 6).

Western blot signal indicates that the maximum relative binding efficiency of EVs to the MPs was $f_{\infty }$ = 0.60 ± 0.01 and is comparable to the standard laboratory assay $f_{\infty }$ = 0.78 ± 0.04 as seen in Figure 6A. The CD63 analysis, performed using an antibody against CD63 as shown in Figure 6B, confirmed the successful capture of extracellular vesicles (EVs) or double-positive membrane fragments, rather than free-floating CD9 protein. The comparable data from both markers confirms most of the signal comes from captured double positive vesicles with little, if any, influence from free floating CD9 protein

To further evaluate the data, an exponential growth model $f=f_{\infty }[1-exp(-st)]$ was fitted for the background corrected integrated WB values using the least square method where a similar model was employed by Petkovica et al. [45]. Here the $f_{\infty }$ is the asymptotic value of the binding efficiency and σ is the binding rate, with results summarized in Table 1.

The data indicate that the majority of EV binding to MPs occur rapidly, within the first 10 min of the reaction. This represents a considerable improvement over to typical laboratory assays, where the binding time of MPs to analytes is expected to be in the range of 1 h to 24 h, as noted by multiple MP protocols [46,47]. Enhanced binding speed in an ON-Chip system compared to traditional antibodies, coupled with the potential for automation, is a key advantage. The entire process can be simplified by setting initial parameters in the flow control system, loading the sample, and removing the magnet, eliminating the need for complex workflows that involve more than 10 manual steps in conventional lab settings. This approach also has the potential to be scaled for use in a simple laboratory instrument, improving efficiency and ease of use. To further improve the microfluidic assay, the overall lengths of the interconnections could be shortened, and the elution buffer could be stored ON-Chip, thereby reducing any elution losses related to passing small volumes of liquid through the tubing. This would improve the particular assay performance but would consequently limit the system flexibility to run other assays.

## 4. Conclusions

In conclusion, a novel microfluidic device, fabricated from cyclic olefin copolymer and off-stoichiometry thiol-ene, has been developed to effectively address common challenges, such as the absorption of lipophilic molecules found in polydimethylsiloxane-based systems. For the analysis of prostate cancer EVs, bioreactor-produced LNCaP-derived EVs were employed. The device enhanced immunomagnetic extracellular vesicle capture, leveraging anti-CD9 nanobodies.

Compared to standard assays, the microfluidic device showed increased EV capture efficiency at flow rates of 100–400 μL/min and incubation times from 1.5 to 3 min. Binding kinetics revealed that anti-CD9 nanobodies facilitate rapid EV capture, and binding saturation is reached in 10 min. The binding efficiency $f_{\infty }$ = 0.6 and 0.62 for the microfluidic device is comparable to $f_{\infty }$ = 0.78 and 0.86 for a lab assay, as indicated by CD9 and CD63 EV biomarker levels in post-capture MP sample. The anit-CD63 antibody analysis confirmed the successful capture of EVs or double-positive membrane fragments, rather than free-floating CD9 protein.

Comparable binding efficiency, paired with reduced manual labor and rapid processing capabilities, demonstrates the potential of the device as a useful tool in clinical diagnostics.

## Figures and Tables

**Table 1:** Asymptotic value of the binding efficiency and the binding rate of integrated WB signals.

	R^2^	$f_{\infty }$	σ
CD9 microfluidics	0.99	0.60	0.53
CD9 Standard assay	0.94	0.78	0.77
CD63 microfluidics	0.99	0.62	0.17
CD63 Standard assay	0.92	0.86	0.60

## Data Availability

The raw data supporting the conclusions of this article will be made available by the authors on request.

## Supplementary Materials

The following supporting information can be downloaded at: https://www.mdpi.com/article/10.3390/polym16243579/s1, Table S1: Antibodies used in Western Blot. Table S2: Correlation matrix of relative mixing index for mixing devices, calculated using Pearson correlation coefficient. The top right cells represent the *p*-value of the correlation coefficient. Figure S1: OSTE-COC passive micromixer design utilized for mixing index testing. Figure S2: Magnetic separation module dimensions in mm. Figure S3: Experimentally tested magnetic configurations using magnetic particles (Dynabeads™). Tests were carried out and results were reported by Cipa et al. Figure S4: Liner regression analysis of integrated intensity for Figure 5, where doted lines represent OFF-Chip MP retention and solid line ON-Chip MP retention. Figure S5: Original uncropped western blot gel images for Figure 3d, Figure S6: Original uncropped western blot gel images for Figure 4a. Figure S7: Original uncropped western blot gel images for Figure 4b. Figure S8: Original uncropped western blot gel images for Figure 4d. Figure S9: Original uncropped western blot gel images for Figure 5. Using CD9 capture and CD63 detection nanobodies. Figure S10: Original uncropped western blot gel images for Figure 5. Using CD9 capture and CD9 detection nanobodies. Figure S11: Original uncropped western blot gel images for Figure 6B. Using CD9 capture and CD63 detection nanobodies. standard assay on the left and microfluidics assay on the right. Figure S12: Original uncropped western blot gel images for Figure 6A. Using CD9 capture and CD9 detection nanobodies. standard assay on the left and microfluidics assay on the right.
