# Supplementary material for: Elucidating Extracellular Vesicle Isolation Kinetics via an Integrated Off-Stoichiometry Thiol-Ene and Cyclic Olefin Copolymer Microfluidic Device
Source: Polymers (Basel). 2024 Dec 21;16(24):3579. doi: 10.3390/polym16243579 (PMC11678796; doi:10.3390/polym16243579)
Supplement: Supplementary file 1 [file polymers-16-03579-s001.zip › polymers-3372474-supplementary.pdf]

Supplementary document

Table S1. Antibodies used in Western Blot

Antibody	Species (primary Ab)	Supplier	Catalogue number	Dilution
Alix Antibody (G-10)	mouse	Santa Cruz Biotechnology	sc-166952	1:1000
Anti-Calnexin antibody	rabbit	Abcam	ab22595	1:2000
Anti-TSG101 antibody [EPR7130(B)]	rabbit	Abcam	ab125011	1:1000
CD63 Antibody (MX-49.129.5)	mouse	Santa Cruz Biotechnology	sc-5275	1:500
CD63 Antibody (SPM524) [HRP]	–	Novus Biologicals	NBP2-34779H	1:1000-1:2000
CD9 nanobody [His_6]	–	–	–	10µg/ml
anti Rabbit – HRP	–	Santa Cruz Biotechnology	sc-2357	1:2000
anti Mouse IgGk – HRP	–	Santa Cruz Biotechnology	sc-516102	1:2000
6x-His Tag Monoclonal Antibody (HIS.H8), HRP	–	Thermo Fisher Scientific	MA1-21315-HRP	1:1000

Validation of design using relative mixing index

To evaluate various designs and the device fabrication process, OSTE-COC devices were created as detailed in the results and discussion section, with channel geometries illustrated in Supplementary Figure 1.

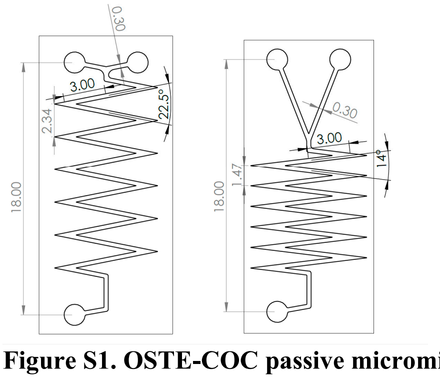

Figure S1. OSTE-COC passive micromixer design utilized for mixing index testing.

After device fabrication, the mixing performance was experimentally assessed using three microfluidic devices, each with seven channels. This assessment involved measuring initial and final concentrations of rhodamine B and deionized water over multiple runs. The mixing index values were determined using the relative mixing index, as described by Ali Hashmi and Jei Xu in 2014.[1] The relative mixing index was calculated using the following formula:

$$RMI = 1 - \frac{\sigma}{\sigma_0} = 1 - \frac{\sqrt{\frac{1}{N}\sum_{i=1}^{N}(I_i - <I>)^2}}{\sqrt{\frac{1}{N}\sum_{i=1}^{N}(I_{oi} - <I>)^2}}$$

σ = standard deviation of pixel intensities across a cross section

σ_0 = standard deviation of the pixel intensities in the unmixed case

N = represents the total number of pixels.

$<I>$ = average of the pixel intensities in the cross section

I_i = local pixel intensity

I_{oi} = local pixel intensity in the unmixed state

Quantification of the geometric parameters was performed using a dataset comprising 54 points. A correlation matrix was then calculated to quantify the strength and direction of linear relationships between variables.

Table S2. Correlation matrix of relative mixing index for mixing devices, calculated using Pearson correlation coefficient. The top right cells represent the p-value of the correlation coefficient.

	Flow rate	Channel size	Angle	RMI
**Flow rate**	1	3.50×10^{-1}	0.79	2.57×10^{-8}
**Channel size**	-0.13	1	0.49	9.00×10^{-5}
**Angle**	0.04	-0.1	1	2.78×10^{-1}
**RMI**	0.67	-0.51	-0.15	1

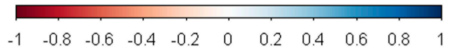

Magnetic separation module dimensions

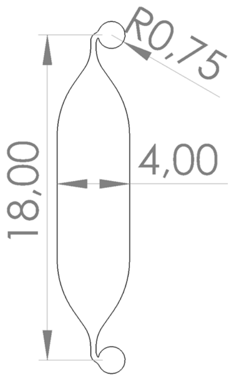

Figure S2. magnetic separation module dimensions in mm.

Magnetic capture module configurations

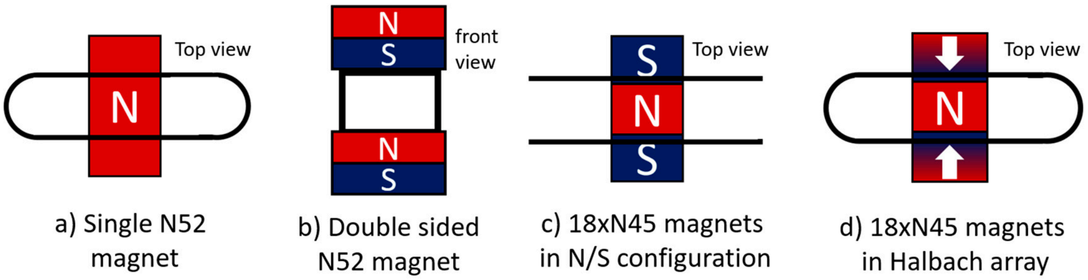

Figure S3. Experimentally tested magnetic configurations using magnetic beads (Dynabeads™). Tests were carried out and results were reported by Cipa et.al.[2]

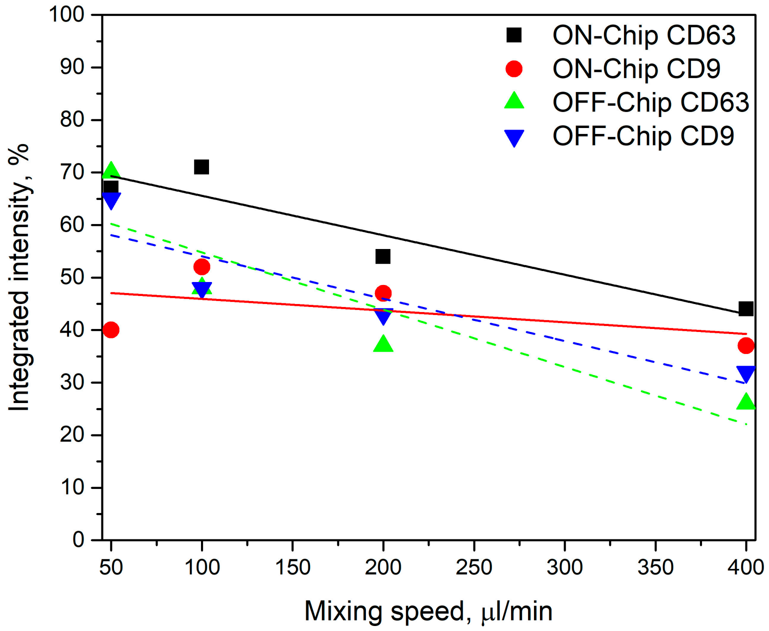

Figure S4, Liner regression analysis of integrated intensity for figure 5, where doted lines represent OFF-chip MP retention and solid line ON-chip MP retention.

Original uncropped images of representative western blots displayed in the manuscript.

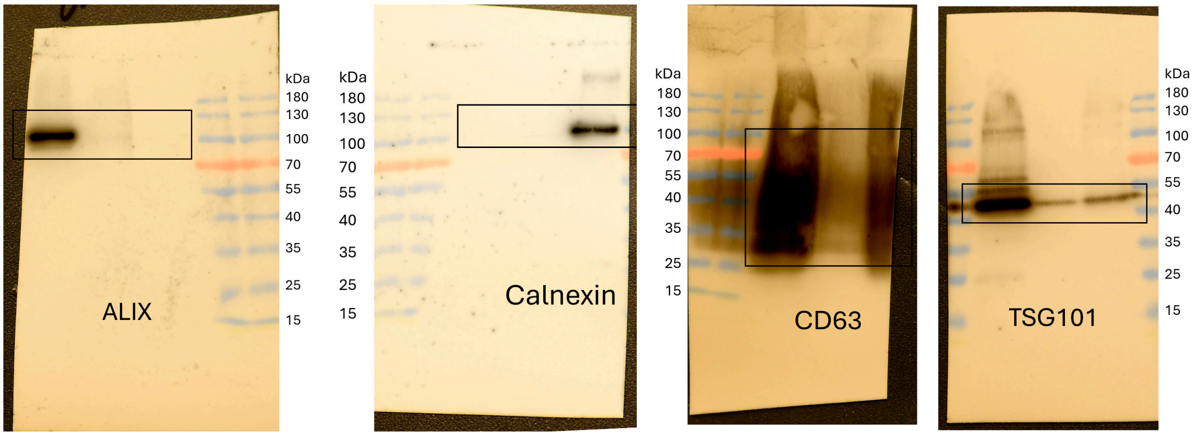

Figure S5, original uncropped western blot images for figure 3D

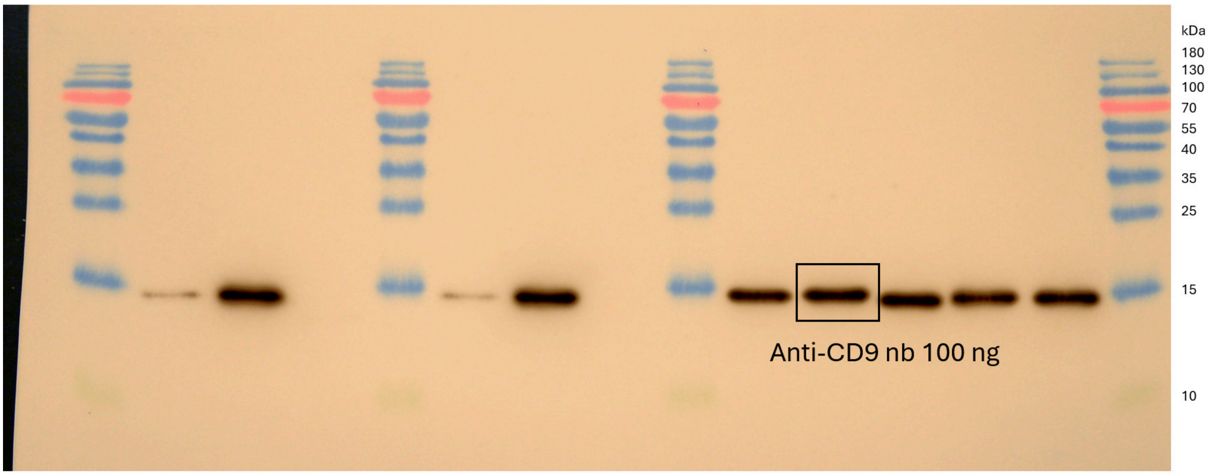

Figure S6, original uncropped western blot images for figure 4A.

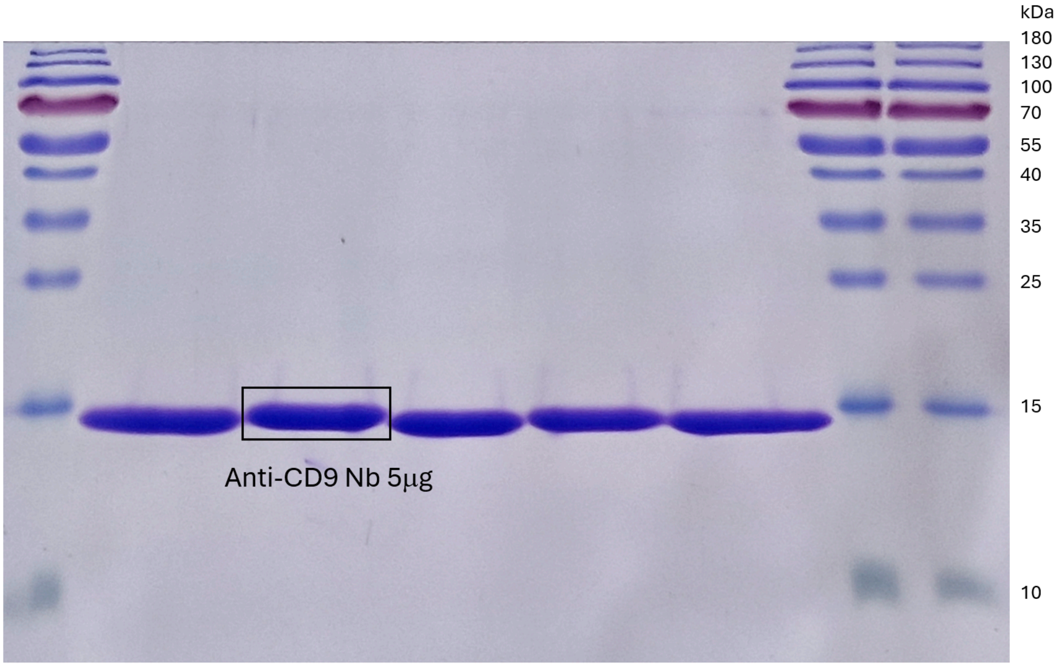

Figure S7, original uncropped Coomassie stained gel blot gel images for figure 4B.

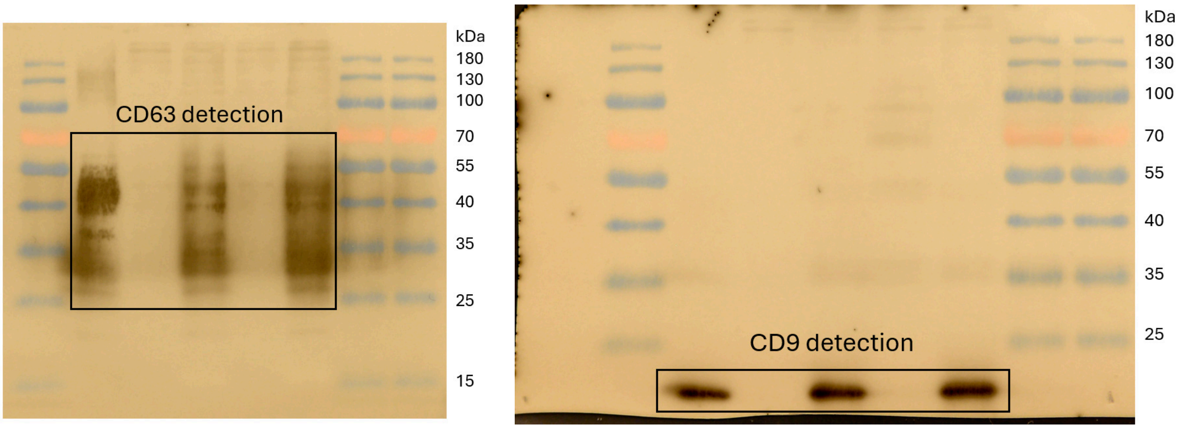

Figure S8, original uncropped western blot images for figure 4D.

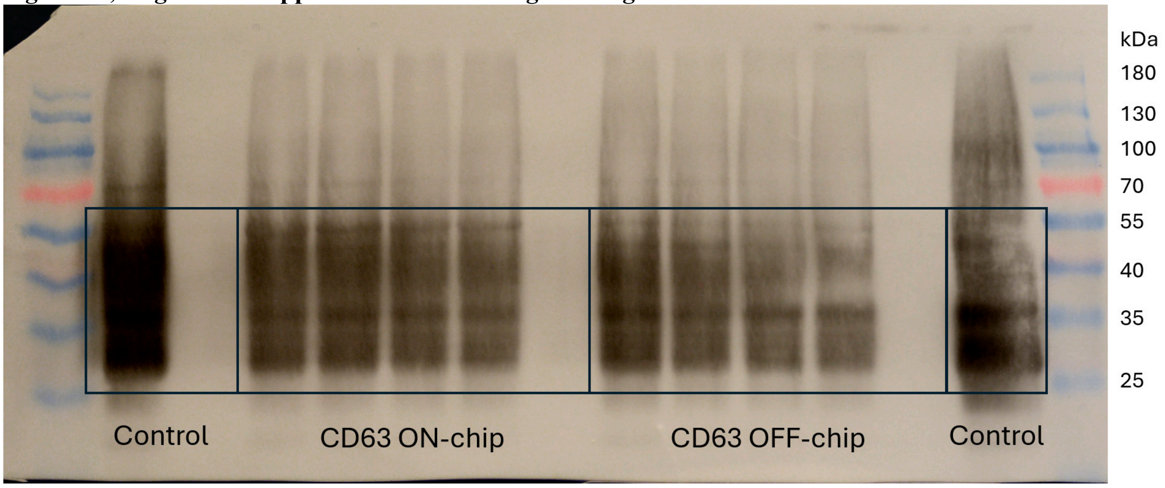

Figure S9, original uncropped western blot images for figure 5. Using CD9 capture and CD63 detection nanobodies.

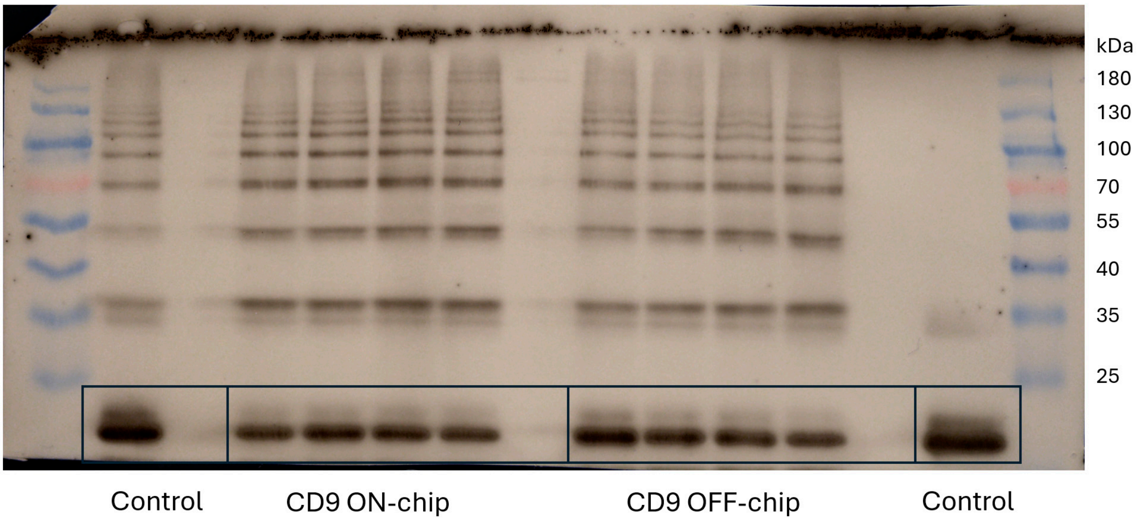

Figure S10, original uncropped western blot images for figure 5. Using CD9 capture and CD9 detection nanobodies.

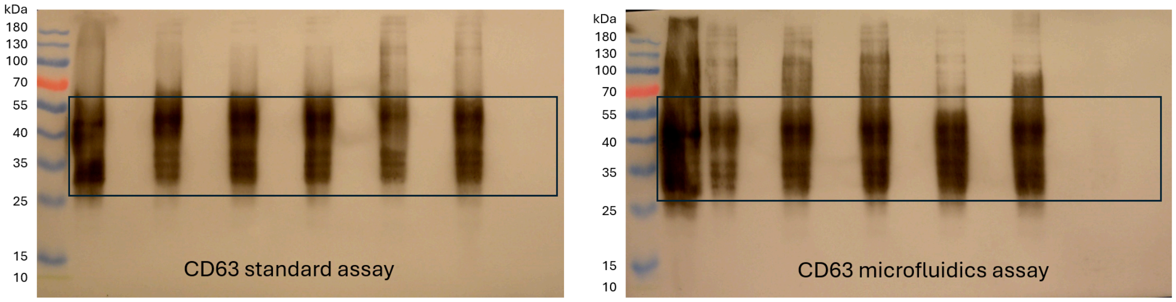

Figure S11, original uncropped western blot images for 6B. Using CD9 capture and CD63 detection nanobodies. standard assay on the left and microfluidics assay on the right.

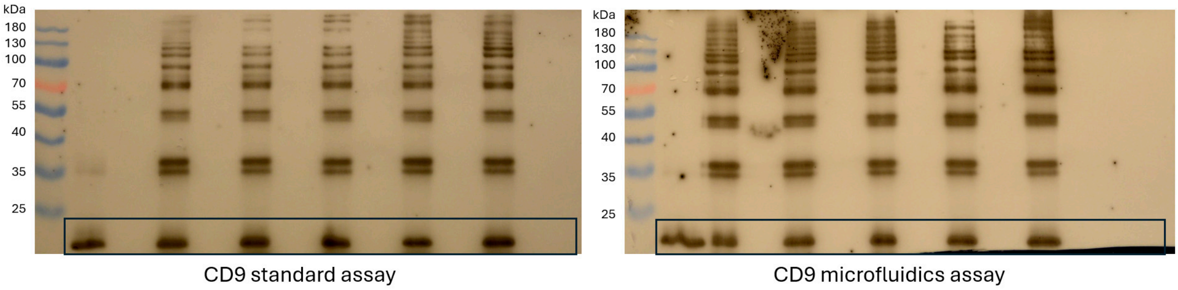

Figure S12, original uncropped western blot images for figure 6A. Using CD9 capture and CD9 detection nanobodies. standard assay on the left and microfluidics assay on the right.

For all western blot images prestained protein Ladder (PageRuler^{TM} 10 to 180 kDa) was used. In figure 8 and figure 10 Higher molecular weight bands are observed due to the presence of multimeric EV-capture nanobodies from anti-CD9 beads, which are also stained with the secondary antibody.

References.

1. Hashmi, A. & Xu, J. On the quantification of mixing in microfluidics. *J Lab Autom* **19**, 488–491 (2014).

2. Cipa, J. *et al.* OSTE DEVICE FOR MAGNETIC PARTICLE CAPTURE. *MicroTAS 2022 - 26th International Conference on Miniaturized Systems for Chemistry and Life Sciences* 919–920 (2022).
